# Potentially Functional Polymorphisms in* POU5F1* Gene Are Associated with the Risk of Lung Cancer in Han Chinese

**DOI:** 10.1155/2015/851320

**Published:** 2015-12-28

**Authors:** Rui Niu, Yuzhuo Wang, Meng Zhu, Yifan Wen, Jie Sun, Wei Shen, Yang Cheng, Jiahui Zhang, Guangfu Jin, Hongxia Ma, Zhibin Hu, Hongbing Shen, Juncheng Dai

**Affiliations:** Department of Epidemiology and Biostatistics, Jiangsu Key Lab of Cancer Biomarkers, Prevention and Treatment, Collaborative Innovation Center for Cancer Personalized Medicine, School of Public Health, Nanjing Medical University, Nanjing 211166, China

## Abstract

POU5F1 is a key regulator of self-renewal and differentiation in embryonic stem cells and may be associated with initiation, promotion, and progression in cancer. We hypothesized that functional polymorphisms in* POU5F1* may play an important role in modifying the lung cancer risk. To test this hypothesis, we conducted a case-control study to explore the association between 17 potentially functional SNPs in* POU5F1* gene and the lung cancer risk in 1,341 incident lung cancer cases and 1,982 healthy controls in a Chinese population. We found that variant alleles of rs887468 and rs3130457 were significantly associated with increased risk of lung cancer after multiple comparison (OR = 1.29, 95% CI: 1.11–1.51, *P*_fdr_ = 0.017 for rs887468; OR = 1.29, 95% CI: 1.10–1.51, *P*_fdr_ = 0.034 for rs3130457, resp.). In addition, we detected a significant interaction between rs887468 genotypes and smoking status on lung cancer risk (*P* = 0.017). Combined analysis of these 2 SNPs showed a significant allele-dosage association between the number of risk alleles and increased risk of lung cancer (*P*_trend_ < 0.001). These findings indicate that potentially functional polymorphisms in* POU5F1* gene may contribute to lung cancer susceptibility in a Chinese population.

## 1. Introduction

Lung cancer is the leading cause of cancer related mortality worldwide. Over 80% of lung cancer can be attributed to cigarette smoking [1]. However, Only 10% to 15% of chronic smokers develop lung cancer, indicating that other factors (e.g., genetic factors) might also play a pivotal role in lung cancer risk [2]. Recently, genome-wide association studies have discovered dozens of loci that are related to lung cancer risk [3–11]. These loci only account for a small fraction of the risk of developing lung cancer due to the stringent screening criteria of GWAS [8]. Thus, an effort on candidate gene strategies might help to explain the missing heritability.

The Pit-Oct-Unc (POU) homeodomain transcription factor, POU5F1 (also known as OCT-3, OCT-4, and OCT 3/4), is a key regulator of self-renewal and differentiation in embryonic stem cells [12–15].* POU5F1* gene expresses in adult human stem cells, immortalized nontumorigenic cells, and tumor cells and cell lines, and its level decreases with the onset of differentiation and loss of pluripotency in these cells [16–18]. According to the cancer stem cell (CSC) dogma, the reactivation of early embryonic stem cell genes such as* POU5F1* in somatic stem cells and/or differentiating progenitor cells may lead to transformation into CSCs, which may result in cancer initiation, promotion, and progression [19–21]. To date, high expression level of POU5F1 has been detected in various types of cancer cells [22, 23]. In particular, Karoubi et al. observed higher levels of expression of* POU5F1* gene and atypical cytoplasmic distribution of POU5F1 in lung adenocarcinoma cell lines, indicating an oncogenic role in lung adenocarcinoma [24]. Polymorphisms in POU class 5 homeobox 1 pseudogene 1 gene (*POU5F1P1*), a highly homologous pseudogene of* POU5F1*, were identified to be associated with the risk of gastric cancer [25]. Therefore, we postulated that potentially functional genetic variation within* POU5F1* might modify the susceptibility to lung cancer. To test this hypothesis, we conducted a case-control study including 1,341 cases and 1,982 controls to investigate the association between functional polymorphisms in* POU5F1* and lung cancer risk.

## 2. Materials and Methods

### 2.1. Study Subjects

This case-control study was approved by the institutional review board of Nanjing Medical University. Cases were recruited from the Cancer Hospital of Jiangsu Province and the First Affiliated Hospital of Nanjing Medical University since 2003. Patients with histopathologically confirmed incident lung cancer were included. Exclusion criteria include having a prior history of other cancers, having metastatic cancer from other sites, or having undergone chemotherapy of radiotherapy. Controls were randomly selected from individuals participating in a community based noninfectious disease screening program in Jiangsu Province during the same time period. The controls were cancer-free and were frequency matched to cases by age and sex. We enrolled 1,341 cases and 1,982 controls in the final set. After providing a written informed consent, participants donated 5 mL venous blood sample and underwent a face-to-face interview that solicited information on participants' demographics (e.g., age and sex) and health related behaviors (e.g., smoking).

Those who had smoked one cigarette or more per day for >1 year were considered as smokers; smokers who had quit smoking for >1 year were defined as former smokers; all others were classified as never smokers [6]. Smoking dosage were measured by pack-years of smoking [(cigarettes per day/20) × smoking years]. In addition, smokers were divided into light and heavy smokers according to the threshold of 25 pack-years.

### 2.2. SNP Selection

All SNPs in the* POU5F1* gene region and 10 kb upstream were screened based on the Han Chinese population (CHB) of the HapMap Project [HapMap Data Rel. 27 Phase II + III]. Minor allele frequency (MAF) ≥ 0.05 was used to filter low-frequency variants. The remaining variants were annotated by SNPinfo Web Server (http://snpinfo.niehs.nih.gov/); 27 SNPs were selected as potentially functional variants. We then performed linkage disequilibrium (LD) analysis with an *r* ^2^ threshold of 0.8; 19 SNPs were retained for genotyping. However, rs1265163 and rs3132517 were excluded because of probe design failure. Therefore, there were 17 SNPs included in the final set (Table 2).

### 2.3. Genotyping

Genomic DNA was extracted from a leukocyte pellet by proteinase K digestion, followed by phenol-chloroform extraction and ethanol precipitation. Illumina Infinium BeadChip (Illumina Inc.) was used for genotyping and GenTrain version 1.0 clustering algorithm in GenomeStudio V2011.1 (Illumina Inc.) for genotype calling. Technicians performing the genotyping were blinded to the case or control status of participants.

### 2.4. Statistical Analysis

Deviation of genotype distribution for each SNP from the Hardy-Weinberg equilibrium was tested by a goodness-of-fit *χ* ^2^. Student's *t* test for continuous variables and *χ* ^2^ test for categorical variables were applied for analyzing distribution differences of demographic characteristics and genotypes between cases and controls. The association between SNPs and lung cancer risk was examined under an additive model using logistic regression to estimate odds ratios (ORs) [26] and 95% confidence intervals (CIs) with adjustment for age, sex, and pack-years of smoking when appropriate. We used *χ* ^2^-based *Q*-test to test the heterogeneity from corresponding subgroups. Multiplicative interactions were tested using a general logistic regression model by applying the equation

(1) $$\begin{array}{c} Y=\beta_{0}+\beta_{G}G+\beta_{E}S+\beta_{GS}\left(\;GS\;\right)+\sum \beta_{i}{Covar}_{i}, \end{array}$$

where *Y* is the logit of case-control status, *G* and *S* are factors (SNP or smoking status), *β*_0_ is constant, *β*_*G*_ and *β*_*S*_ are the main effects of factors *G* and *S*, respectively. And *β*_*GS*_ is the interaction term. Covar_*i*_ denote covariates for adjustment, including age and sex.

All analyses were performed using R software (version 3.1.1, The R Foundation for Statistical Computing, http://www.cran.r-project.org/).

## 3. Results

The geographic characteristics of participants were summarized in Table 1. The distributions of age (*P* = 0.980) and sex (*P* = 0.179) were comparable between cases and controls. However, cases have larger proportions of smokers and heavy smokers than controls (*P* < 0.001). 481 (35.87%) cases had squamous cell carcinoma and 860 (64.13%) cases had adenocarcinoma.

The call rates of genotyping for 17 SNPs were all above 98% (Table 2). The observed genotypes for these SNPs were in agreement with Hardy-Weinberg equilibrium among controls. The variant alleles of 2 SNPs (rs887468 and rs3130457) were significantly associated with increased risk of lung cancer after multiple comparison (OR = 1.29, 95% CI: 1.11–1.51, *P*_fdr_ = 0.017 for rs887468; OR = 1.29, 95% CI: 1.10–1.51, *P*_fdr_ = 0.034 for rs3130457, resp.) (Tables 2 and 3). However, no significant associations were detected from other SNPs.

To further examine the associations of these 2 SNPs with lung cancer risk, we conducted stratified analyses within subgroups according to selected variables (Table 4). As a whole, the devastating effect of variant alleles was more pronounced in elder age group (>60), males, never smokers, and adenocarcinoma patients. No significant heterogeneity was found for ORs and their 95% CIs between different subgroups. We also explored interaction between these SNPs and smoking status. As shown in Table 5, an interaction existed between the genotype of rs887468 and smoking status (*P* for multiplicative interaction = 0.017). However, no significant multiplicative interaction was observed for rs3130457 (data not shown). Furthermore, we investigated cumulative effect of these 2 risk alleles on lung cancer and observed significant allele-dosage association between number of risk alleles and lung cancer risk (*P*_trend_ < 0.001). People who carried one or two risk alleles were 1.33 times more likely to develop lung cancer than those who carried no risk allele, and those with more than two risk alleles were 1.46 times more likely to develop lung cancer. These data indicated that those who carried more risk alleles had a higher risk of lung cancer.

## 4. Discussion

In the current study, we explored the association of 17 potentially functional SNPs in* POU5F1* gene with the development of lung cancer in 1,341 cases and 1,982 healthy controls. We found that the variant allele of rs887468 and rs1310457 were associated with increased lung cancer risk. To the best of our knowledge, it is the first association study of polymorphisms in the* POU5F1* gene and lung cancer, which provides more evidence for understanding the role of* POU5F1* in lung cancer risk.

POU5F1, a transcription factor, is involved in regulation of pluripotent state of stem cells, and its level decreases with the onset of differentiation in these cells [15–18]. Amini et al. have found that cancer cell lines and cancer tissues had significantly higher expression levels of early embryonic stem cell genes, including* POU5F1*,* SOX2*, and* CD133* [21]. In the hypoxia conditions, POU5F1 can promote* CD133* expression in the lung cancer cells, which is a specific cell surface marker for cancer stem cells [27]. In addition, parallel elevated expression of* POU5F1* and* Nanog* in lung adenocarcinoma (LAC) increases the percentage of* CD133*-expressing subpopulation, enhances drug resistance, and promotes epithelial-mesenchymal transition (EMT); coexpression also activates* Slug* and enhances the tumor-initiating capability of LAC [28, 29]. Furthermore, knockdown of* POU5F1* impeded tumorigenic and metastatic ability and reversed the EMT process of lung adenocarcinoma [28, 29]. For squamous cell carcinoma, Chen et al. measured* POU5F1* expression in nonsmall cell lung cancer tissues and found that although the proportion of squamous cell carcinoma tissues which have elevated expression was smaller than that of adenocarcinoma, the increased expression of* POU5F1* was associated with poor differentiation of cancer cells and shorter overall survival in both histologic subtype of lung cancer [30]. Taken together,* POU5F1* expression is crucial for the self-renewal and oncogenic potentials of lung cancer stem cells. Our findings further demonstrated that* POU5F1* might be a susceptibility gene of lung cancer, which was consistent with the observations mentioned above.

The two SNPs, rs887468 and rs3130457, are located in the upstream region from the transcription starting site of* POU5F1* gene. According to a web-based SNP analysis tool, SNPinfo (http://snpinfo.niehs.nih.gov/), the two SNPs are all potential transcriptor binding sites and the SNP rs887468 may influence an exonic splicing enhancer [31] or exonic splicing silencer (ESS) (Supplemental Table 1) (see Supplementary Material available online at http://dx.doi.org/10.1155/2015/851320). Variations in ESE or ESS can be involved in disruptions of balanced interplay of ESE- and ESS-binding proteins, which thereby results in missplicing and causes deficiency in expression products of nearby genes [31–34]. We speculate that variant genotype of rs887468 might lead to alternative splicing events and disequilibrium for different isoforms of* POU5F1*, suggesting a biological plausible mechanism for lung cancer risk. Variation in rs887468 may influence its interactions with transcription factors such as Myc-associated zinc-finger protein (MAZ) (as predicted by RegulomeDB (http://www.regulomedb.org/)).* MAZ* gene normally expresses in the lungs [35]. It is responsible for regulation of oncogene transcription from promoter [36]. One possible explanation for the association between rs887468 and lung cancer risk might be that the variant genotype alters interactions of the loci with transcription factors and results in aberration in function of* POU5F1* gene and elicits procedure of carcinogenesis.

Based on an online tool, DNase-seq and RegulomeDB (http://www.regulomedb.org/), rs887468 and rs3130457 fall into DNase I peaks. DNase I hypersensitivity regions are potential genetic regulatory loci and correlate with binding sites of sequence-specific DNA-binding proteins [37]. This suggests a possible mechanism for the effect of these variants on lung cancer risk. Additionally, a multiplicative interaction was observed between rs887468 in* POU5F1* gene and smoking status. The interaction result indicated that the harmful effect of variant allele of rs887468 was weaker among current smokers. However, our results are very preliminary; further experimental studies are warranted to uncover the underling mechanism of these observations.

In summary, our case-control study reported 2 potential functional SNPs in* POU5F1* gene that may affect the risk for lung cancer in a Chinese population. Given that our results are very preliminary, more large scale studies are required to validate our findings in diverse ethnic populations and clarify the molecular basis behind these observations.

## Supplementary Material

To search for potentially functional variation, common SNPs in POU5F1 were annotated by SNPinfo Web Server (). Among the 17 genotyped SNPs, 14 SNPs are located in transcription factor binding sites, 7 SNPs are exonic splicing enhancer (ESE) or exonic splicing silencer (ESS). Functional annotation of the 17 genotyped SNPs are summarized in Supplementary Table 1.

## Figures and Tables

**Table 1:** Distributions of select variables in lung cancer cases and cancer-free controls.

Variables	Case (*N* = 1,341)	Control (*N* = 1,982)	*P*
Age (mean ± sd.)	61.06 ± 10.15	61.32 ± 11.07	0.473
≤60	596 (44.44%)	883 (44.55%)	0.980
>60	745 (55.56%)	1099 (55.45%)	
Gender			
Male	949 (70.77%)	1358 (68.52%)	0.179
Female	392 (29.23%)	624 (31.48%)	
Smoking status			
Current	634 (47.28%)	876 (44.20%)	**<0.001**
Former	185 (13.80%)	86 (4.34%)	
Never	522 (38.92%)	1020 (51.46%)	
Pack-year (py.)			
≤25	774 (57.72%)	1505 (75.93%)	**<0.001**
>25	567 (42.28%)	477 (24.07%)	
Histology type			
Squamous cell carcinoma	481 (35.87%)		
Adenocarcinoma	860 (64.13%)		

**Table 2:** Summary of associations between 17 SNPs in selected *POU5F1* gene with lung cancer risk.

SNP	Allele^a^	Case^b^	Control^b^	Call rate (%)	MAF (case/control)^c^	HWE^d^	OR (95% CI)^e^	*P* ^e^	*P* ^f^
**rs887468**	**G/A**	**997/322/22**	**1588/368/26**	**100.00**	**0.14/0.11**	**0.35**	**1.29 (1.11–1.51)**	**0.001**	**0.017**
**rs3130457**	**A/G**	**1030/293/18**	**1626/335/21**	**100.00**	**0.12/0.10**	**0.43**	**1.29 (1.10–1.51)**	**0.002**	**0.034**
rs12215963	A/G	1102/221/8	1694/254/9	98.95	0.09/0.07	1.00	1.26 (1.05, 1.53)	0.015	0.255
rs885948	A/G	321/665/355	530/1000/452	100.00	0.51/0.48	0.65	1.13 (1.02, 1.25)	0.022	0.374
rs4713438	G/A	978/335/28	1391/552/39	100.00	0.15/0.16	0.07	0.90 (0.78, 1.04)	0.169	1.000
rs2269713	A/G	875/411/55	1337/579/66	100.00	0.19/0.18	0.70	1.09 (0.96, 1.24)	0.181	1.000
rs1052989	G/A	556/611/174	772/931/279	100.00	0.36/0.38	0.96	0.93 (0.84, 1.04)	0.201	1.000
rs885950	A/C	463/636/241	722/930/322	99.73	0.42/0.40	0.45	1.07 (0.96, 1.18)	0.208	1.000
rs879882	A/G	342/676/323	553/964/465	100.00	0.49/0.48	0.26	1.06 (0.96, 1.18)	0.227	1.000
rs2394882	C/A	617/577/147	851/914/217	100.00	0.32/0.34	0.23	0.94 (0.84, 1.04)	0.239	1.000
rs9468877	G/A	1141/193/7	1718/256/8	100.00	0.08/0.07	0.86	1.12 (0.92, 1.35)	0.275	1.000
rs1108746	C/A	1097/233/11	1607/356/19	100.00	0.10/0.10	1.00	0.95 (0.80, 1.12)	0.541	1.000
rs1265156	C/A	589/579/173	835/900/247	100.00	0.34/0.35	0.84	0.97 (0.88, 1.08)	0.597	1.000
rs887464	G/A	487/628/226	684/963/335	100.00	0.40/0.41	0.93	0.97 (0.88, 1.08)	0.625	1.000
rs3130503	G/A	791/485/65	1154/726/102	100.00	0.23/0.23	0.42	0.98 (0.87, 1.11)	0.773	1.000
rs3094188	A/C	834/452/55	1250/650/82	100.00	0.21/0.21	0.89	1.02 (0.90, 1.15)	0.775	1.000
rs887466	G/A	403/656/281	587/981/413	99.94	0.45/0.46	0.93	1.00 (0.90, 1.10)	0.955	1.000

^a^Major/minor allele. ^b^Major homozygote/heterozygote/rare homozygote between cases and controls. ^c^Minor allele frequency. ^d^Hardy-Weinberg equilibrium test among controls.

^e^Logistic regression with adjustment for age, sex, and pack-year of smoking in additive model.

^f^ *P* values of false discovery rate (FDR).

**Table 3:** Associations between 2 functional SNPs in *POU5F1* gene with lung cancer risk.

Genotype	Case (*N* = 1,341)	Control (*N* = 1,982)	OR (95% CI)^b^	*P* ^b^
rs887468 (G>A)^a^				
GG	997	1588	1.00	
GA	322	368	1.33 (1.12–1.59)	**0.001**
AA	22	26	1.39 (0.78–2.49)	0.269
Additive model	1341	1982	1.29 (1.11–1.51)	**0.001**
rs3130457 (A>G)^a^				
AA	1030	1626	1.00	
AG	293	335	1.31 (1.10–1.57)	**0.003**
GG	18	21	1.44 (0.76–2.76)	0.265
Additive model	1341	1982	1.29 (1.10–1.51)	**0.002**

^a^Major allele > Minor allele. ^b^Logistic regression with adjustment for age, gender, and pack-years of smoking.

**Table 4:** Stratified analysis on the associations of rs887468 and rs3130457 in *POU5F1* with lung cancer risk.

Characteristics	rs887468 (G>A)				*P*_het_^c^	rs3130457 (A>G)				*P*_het_^c^
	Case^a^	Control^a^	OR (95% CI)^b^	*P* ^b^		Case^a^	Control^a^	OR (95% CI)^b^	*P* ^b^	
Age					0.093					0.079
≤60	450/136/10	689/185/9	1.11 (0.88–1.40)	0.392		463/124/9	705/170/8	1.09 (0.85–1.38)	0.498	
>60	547/186/12	899/183/17	1.45 (1.18–1.79)	**<0.001**		567/169/9	921/165/13	1.46 (1.17–1.82)	**<0.001**	
Gender					0.749					0.398
Male	712/222/15	1100/241/17	1.32 (1.09–1.59)	**0.004**		734/200/15	1126/219/13	1.35 (1.11–1.64)	**0.003**	
Female	285/100/7	488/127/9	1.25 (0.95–1.65)	0.105		296/93/3	500/116/8	1.16 (0.87–1.56)	0.312	
Smoking status					0.170					0.455
Current	493/133/8	697/166/13	1.08 (0.86–1.36)	0.511		503/124/7	715/152/9	1.14 (0.89–1.44)	0.296	
Former	127/52/6	66/19/1	1.54 (0.9–2.64)	0.114		135/44/6	69/16/1	1.53 (0.87–2.68)	0.136	
Never	377/137/8	825/183/12	1.44 (1.14–1.81)	**0.002**		392/125/5	842/167/11	1.37 (1.07–1.75)	**0.011**	
Pack-years					0.331					0.680
0	377/137/8	825/183/12	1.44 (1.14–1.81)	**0.002**		392/125/5	842/167/11	1.37 (1.07–1.75)	**0.011**	
0–25	193/53/6	393/85/7	1.21 (0.86–1.70)	0.270		200/47/5	403/76/6	1.19 (0.83–1.70)	0.336	
>25	427/132/8	370/100/7	1.11 (0.86–1.45)	0.422		438/121/8	381/92/4	1.18 (0.90–1.56)	0.231	
Histology type					0.198					0.285
Squamous cell carcinoma	370/103/8	1588/368/26	1.12 (0.88–1.42)	0.358		381/92/8	1626/335/21	1.14 (0.88–1.46)	0.319	
Adenocarcinoma	627/219/14	1588/368/26	1.36 (1.15–1.62)	**<0.001**		649/201/10	1626/335/21	1.35 (1.13–1.62)	**0.001**	

^a^Wild-type homozygote/heterozygote/variant homozygote. ^b^Adjusted for age, gender, and pack-years of smoking where appropriate in additive model. ^c^ *P* for heterogeneity.

**Table 5:** The interaction between rs887468 and smoking status on lung cancer risk.

Smoking status	rs887468			OR (95% CI)^a^	*P* ^a^
	Genotype	Case	Control		
Nonsmokers	GG	504	891	1.00	
Nonsmokers	GA/AA	203	215	1.67 (1.34–2.08)	**<0.001**
Smokers	GG	493	697	1.21 (1.01–1.46)	**0.039**
Smokers	GA/AA	141	179	1.35 (1.04–1.76)	**0.024**
*P* for multiplicative interaction					**0.017**

^a^ *P* value of interaction analysis between rs887468 and pack-years of smoking on lung cancer risk with adjustment for age and gender.
