# Supplementary material for: Potentially Functional Polymorphisms in POU5F1 Gene Are Associated with the Risk of Lung Cancer in Han Chinese
Source: Biomed Res Int. 2015 Dec 28;2015:851320. doi: 10.1155/2015/851320 (PMC4707383; doi:10.1155/2015/851320)
Supplement: Supplementary file 1 — To search for potentially functional variation, common SNPs in POU5F1 were annotated by SNPinfo Web Server (). Among the 17 genotyped SNPs, 14 SNPs are located in transcription factor binding sites, 7 SNPs are exonic splicing enhancer (ESE) or exonic splicing silencer (ESS). Functional annotation of the 17 genotyped SNPs are summarized in Supplementary Table 1. [file 851320.f1.pdf]

SUPPLEMENTARY MATERIALS

Potentially functional polymorphisms in *POU5F1* gene are associated with the risk of lung cancer in Han Chinese

Rui Niu, Yuzhuo Wang, Meng Zhu, Yifan Wen, Jie Sun, Wei Shen, Yang Cheng, Jiahui Zhang, Guangfu Jin, Hongxia Ma, Zhibin Hu, Hongbing Shen, Juncheng Dai

Supplementary Table 1. Functional annotation of 17 functional SNPs in *POU5F1* gene according to SNPinfo

SNP	TFBS [a]	Splicing (site)	Splicing (ESE or ESS) [b]	Splicing (abolish domain)	miRNA (miRanda)	miRNA (Sanger)	nsSNP [c]	Stop Codon
rs1052989	Y	--	--	--	--	--	--	--
rs1108746	Y	--	--	--	--	--	--	--
rs12215963	Y	--	--	--	--	--	--	--
rs1265156	Y	--	Y	--	--	--	--	--
rs2269713	Y	--	--	--	--	--	--	--
rs2394882	--	--	Y	--	--	--	--	--
rs3094188	Y	--	--	--	--	--	--	--
rs3130503	--	--	Y	--	--	--	--	--
rs4713438	Y	--	--	--	--	--	--	--
rs879882	Y	--	--	--	--	--	--	--
rs885948	Y	--	--	--	--	--	--	--
rs887464	Y	--	Y	--	--	--	--	--
rs887466	--	--	Y	--	--	--	--	--
rs887468	Y	--	Y	--	--	--	--	--
rs885950	Y	--	--	--	--	--	--	--
rs3130457	Y	--	--	--	--	--	--	--
rs9468877	Y	--	Y	--	--	--	--	--

[a] Transcription factor binding sites;

[b] ESE: Exonic splicing enhancer; ESS: Exonic splicing silencer;

[c] Non-synonymous coding SNPs.
